# Comparative Aroma Profile Analysis and Development of a Sensory Aroma Lexicon of Seven Different Varieties of *Flammulina velutipes*

**DOI:** 10.3389/fnut.2022.827825

**Published:** 2022-04-28

**Authors:** Ruijuan Wang, Yueyan Zhang, Huan Lu, Jianyu Liu, Chunyan Song, Zhen Xu, Hui Yang, Xiaodong Shang, Tao Feng

**Affiliations:** ^1^Key Laboratory of Applied Mycological Resources and Utilization, Ministry of Agriculture, National Research Center for Edible Fungi Biotechnology and Engineering, Shanghai, China; ^2^Shanghai Key Laboratory of Agricultural Genetics and Breeding, Institute of Edible Fungi, Shanghai Academy of Agricultural Sciences, Shanghai, China; ^3^School of Perfume and Aroma Technology, Shanghai Institute of Technology, Shanghai, China

**Keywords:** *Flammulina velutipes* (*F. velutipes*), gas chromatography-mass spectrometry (GC-MS), sensory, lexicon, aroma

## Abstract

*Flammulina Velutipes* (*F. velutipes*) is widely planted all over the world and is rich in nutrients, which is of great benefit to the human body. However, the research on the aroma of *F. velutipes* is relatively rare, which limits the application of *F. velutipes* in deep processing, resulting in a single product and edible method of *F. velutipes*. The purpose of this study was to find out the aroma compounds contributing to the sensory properties of *F. velutipes* to promote the application of different varieties of *F. velutipes* in deep processing. Aromas of 7 species of *F. velutipes* were described and evaluated by sensory evaluation experiment. The volatile compounds in seven kinds of *F. velutipes* were detected by headspace solid-phase microextraction combined with gas chromatography-mass spectrometry (GC-MS). A total of 74 volatile compounds were found, including 23 alcohols, 5 aldehydes, 2 phenols, 1 acid, 16 esters, 7 ketones, 1 ether, 13 hydrocarbons, 1 sulfide, 1 acyl compound, and 4 heterocyclic compounds. It was also found that the sensory evaluation results of sample F, C, and E had a high correlation with the content of compound, and the correlation between sample B and sample A was also high. A lexicon for describing aroma attributes of *F. velutipes* was developed and they could be grouped into categories, such as fruity (apple-like, banana-like, cucumber-like, citrus-like and berry-like), alcoholic (whisky-like, fermented fruit-like), milky (creamy-like), floral (hyacinth-like, phoenix-like, iris-like and mint-like), sulfurous (onion-like), and musty (mud-like). This research will provide a theoretical basis for the future study of *F. velutipes* aroma and the development and application of *F. velutipes* products.

## Introduction

*Flammulina Velutipes* (*F. velutipes)* is a kind of fungus with rich-nutritional value, which is planted all over the world and has a long cultivation history in China (1). In recent years, *F. velutipes* is favored by consumers because of its delicious taste and excellent taste (2). *F. velutipes* contains protein, various amino acids, unsaturated fatty acids, crude fiber, vitamins, calcium, magnesium, zinc, potassium, and other nutrients (3), which has been selected as the best vegetable in the World Health Organization's best food group (4). *F. velutipes* can effectively enhance the living activity of the body, promote the metabolism, facilitate the absorption and utilization of various nutrients in food, and benefit growth and development. Simultaneously, it can inhibit the increase of blood lipids, lower cholesterol, and prevent hyperlipidemia, thus reducing the occurrence of cardiovascular diseases (5).

Most studies on *F. velutipes* currently focus on nutritional value and shelf life, with few studies focusing on aroma characteristics of *F. velutipes* (6). *F. velutipes* is a single product, which can be eaten directly in most cases. Studying the aroma characteristics of different varieties of *F. velutipes* and finding out the differences of aroma of different varieties of *F. velutipes* can provide a theoretical basis for deep processing of *F. velutipes* to different products. In this experiment, Solid-phase microextraction (SPME) was used to extract volatile compounds from different varieties and analyzed by gas chromatography-mass spectrometry (GC-MS). The sensory evaluation established the aroma evaluation system of *F. velutipes*, and Partial Least Squares Regression (PLSR) was used to determine the aroma compounds of *F. velutipes*.

Aroma lexicons were widely used to record and describe the sensory properties of foods (7). Aroma lexicon had been developed to compare the aroma characteristics of beef processed in different ways (8). The aroma characteristics of pomegranate juice were summarized through aroma lexicon, so as to promote the development of new products and enable researchers to have a deeper understanding of their aroma characteristics (9). Therefore, it is of great significance to summarize the aroma characteristics of *F. velutipes* in processing.

The objectives of this research were (1) to develop a lexicon of different types of *F. velutipes*, and (2) to study the relationship between sensory analysis and aroma-active compounds to identify the volatile compounds that contribute mainly to the characteristic aroma of different *F. velutipes* varieties. This research can promote the application of different varieties of *F. velutipes* in the deep processing of food.

## Materials and Methods

### Samples

The strains, origins and appearances of the seven samples of Flammulina velutipes has been shown in Table 1 (numbered A, B, C, D, E, F, G, respectively).

**Table 1:** *Flammulina Velutipes* strains, origin, and fruit body morphology.

**NO**.	**Strain**	**Origin**	**Fruit body morphology**
A	No.61523	Institute of Edible Fungi, Shanghai Academy of Agricultural Sciences	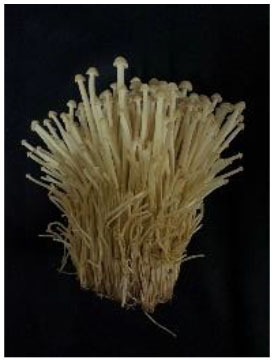
B	No.61525	Institute of Edible Fungi, Shanghai Academy of Agricultural Sciences	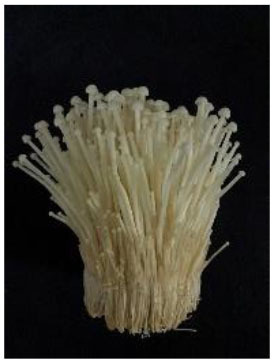
C	No.61486	Institute of Edible Fungi, Shanghai Academy of Agricultural Sciences	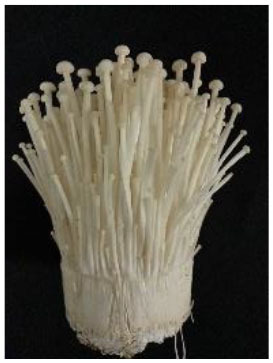
D	No.61529	Institute of Edible Fungi, Shanghai Academy of Agricultural Sciences	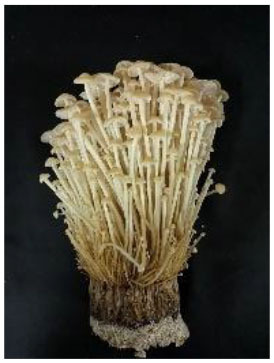
E	No.61530	Institute of Edible Fungi, Shanghai Academy of Agricultural Sciences	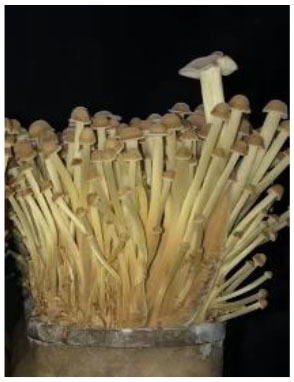
F	No.61490	Institute of Edible Fungi, Shanghai Academy of Agricultural Sciences	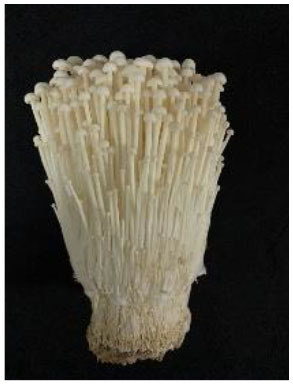
G	CK	Institute of Edible Fungi, Shanghai Academy of Agricultural Sciences	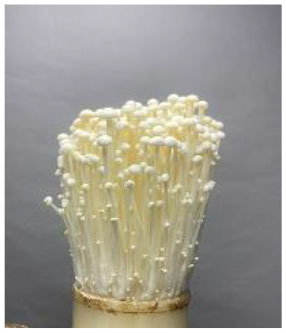

#### Sensory Assessment of Seven Kinds of *F. velutipes*

##### Selection of Sensory Panel

We called to the 100 consumers who enjoy *F. velutipes* by means of online recruitment. Using questionnaire and test method for screening of olfactory relatively sensitive 50. The test sheets were listed in Table 2.

**Table 2:** Sample table in the test sheet for the odor sensitive test.

**Code**	**Dirty**	**Bacterial-aroma**	**Liquor**	**Creamy**	**Apple**	**Mold**	**Banana**	**Green, grass**	**Fresh**	**Mushroom**	**Iris**	**Brake**	**Vegetable**	**Milk**	**Wine**
612															
346															
128															
573															
209															
430															
794															

##### Collection of Descriptive Words

Free choice profiling (FCP) was used to evaluate sensory characteristics of the raw *F. velutipes* samples of seven cultivars. As many sensory descriptive words as possible were recorded in person by the panel for subsequent discussion. A total of fifty consumers were selected to describe the *F. velutipes* existing in the market, 15 aroma descriptions were collected: dirty, bacterial aroma, liquor, creamy, apple, mold, banana, green grass, fresh, mushroom, iris, brake, vegetable, milk, and wine.

##### Determination of Descriptive Words

Total 10 professional sensory evaluators, including 5 men and 5 women from Shanghai Institute of Technology, Shanghai, China, were selected from 30 persons (aged from 22 to 32 years) in terms of (ISO 8586-1, 2012) for selection, training, and monitoring. Out of the 15 descriptors selected in the previous part, ten professional evaluators screened, evaluated, and summarized. The following six descriptors were selected as descriptive indicators of sensory evaluation, such as fruity, alcoholic, milky, floral, sulfurous, and musty.

##### Panelists Training

The sensory evaluation experiment was carried out in accordance with ISO 8586. All work with human subjects performed here was reviewed and approved by the Shanghai Institute of Technology Institutional Review Board(RIB). The panelists requested 8 h of training, composed of four 2-h sessions conducted over 2 weeks.

##### Training Session I

The purpose of this phase is to find representative compounds of the characteristic aromas. Representative aroma compounds were determined by references: fruity: ethyl 2-methylbutyrate (>99%); alcoholic: isoamyl alcohol (>99%); milky: acetoin(>99%); floral: phenylacetaldehyde(>99%); sulfurous: dimethyl disulfide(>99%); musty: geosmin (>99%). These compounds were made into solutions with dilution ratios of 1:10, 1:20; 1:40, 1:80, and 1:160, respectively.

##### Training Session II

Total six representative aroma compounds were diluted at a certain concentration (10 μg/kg~100 mg/kg), each sample was sniffed by 10 sensory evaluators and scored from 0 to 5 (0: no smell, 1: very weak, 2: weak, 3: medium, 4: strong, and 5: very strong).

##### Sensory Assessment

Each sample (10.0 g) was placed in a 30 mL sensory plastic cup with a lid in individual sensory booths under dim light to mask the color difference between samples, noted with a random number. Then the panelists were asked to give aroma intensity scores of each sensory attribute at room temperature (21°C). Each panelist was asked to have a rest for sensory recovery between each set of seven different samples. The process was repeated in triplicate.

### Isolation Method

The extraction fiber was aged at 250°C for 0.5 h. Total seven kinds of *F. velutipes* were frozen into powder by liquid nitrogen, then pulverize powder with a crusher into powder [200 mesh, CS-700 crusher (600Y, Yongkang baoou Hardware Products Co., Ltd, Zhejiang, China)]. Then 5 g powdered sample was added to the 20 ml headspace bottle, adsorbed in a water bath at 60°C for 40 min, and then analyzed by GC-MS.

### GC-MS Conditions

Gas chromatography conditions: The sample entered the DB-WAX quartz capillary column (15 m × 0.53 mm, 0.5 m) as a gas. The carrier gas was He (purity ≥ 99.999%), and the programmed temperature was as follows: the inlet temperature was 230°C, the initial column temperature was 35°C, maintained for 5 min, then rose to 150°C at 3°C/min, then rose to 230° at 8°C/min, and stayed for 2 min. Carrier gas (He) flow 1 mL/min.

MS conditions: Ionization mode EI, electron energy 70 eV, transmission line temperature 250°C, ion source temperature 230°C, mass scanning range 30 ~ 550 m/z, scanning interval 1 s.

The volatiles were identified in terms of mass spectra, and odor activity value (OAV). The reference is from Technology (NIST) 11 and database: https://webbook.nist. The content of each compound is calculated by the peak area of the inner standard 2-octanol.

### Statistical Analysis

Analysis of variance (ANOVA) tests were performed on sensory and chemical data through Statistics 8.1(SAS Institute Inc., Cary, USA). To understand the main differences more clearly between *F. velutipes* varieties, different samples were compared with partial least squares by the Unscrambler X 10.4(CAMO SOFTWARE AS, Oslo, NORWAY).

## Results and Discussion

### Sensory Analysis

**Figure 1:**
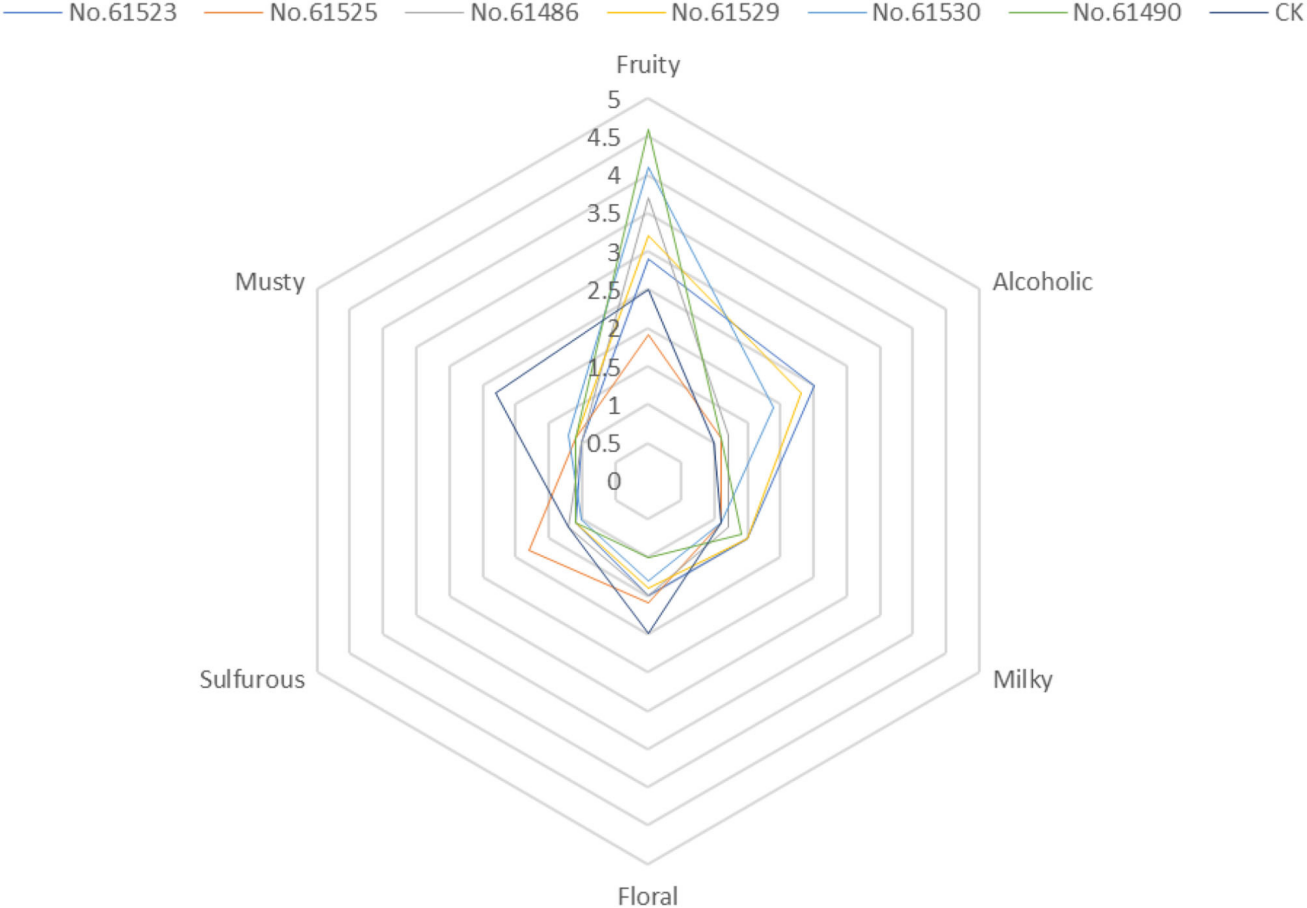
Sensory spider plot by sensory evaluation of seven kinds of *F. velutipes*. Each value represents the average aroma intensity score by 10 panelists with three replications.

Statistics on the sensory evaluation results, as shown in the sensory radar chart of Figure 1. Overall, fruity is the highest in all samples. The alcoholic is the highest in the No. 61,530 sample. The five *F. velutipes* samples are different in the score of the milky. The floral and musty are highlighted in the sample G, and the sulfurous aroma is important in the No. 61,525 sample.

### Compounds Detected by GC-MS

As shown in Table 4, a total of 74 volatile compounds were found, including 23 alcohols, 5 aldehydes, 2 phenols, 1 acid, 16 esters, 7 ketones, 1 ether, 13 hydrocarbons, 1 sulfide, 1 acyl compound, and 4 heterocyclic compounds.

#### Alcoholic

Alcoholic compounds have a larger threshold and contribute less to aroma. There are three kinds of alcohol compounds with OAV >1 in all samples in this experiment, which are 2-methyl-1-butanol, 3-methyl-1-butanol, and 1-hexanol. The aroma characteristics of 2-methyl-1-butanol, are described as whisky aroma (10). The odor of 3-methyl-1-butanol is unpleasant (11). Many fruits and tea leaves contain 1-hexanol, which is often added to essential oils in the industry (12).

#### Aldehydes

Aldehydes are a kind of volatile compounds with a low threshold, which contribute greatly to the aroma of food. The five kinds of aldehydes were detected in this experiment, namely, benzaldehyde, benzene acetaldehyde, (E,E)-2,4-Decadienal, Phenol, and 2-nonenal. Benzaldehyde is an aromatic substance with a pleasant aroma (13). In this experiment, benzaldehyde was only detected in A and E samples, and the OAV was <1. Benzene acetaldehyde is a kind of fatty aldehyde with unstable properties, which has the fragrance of flowers like hyacinth. The OAV of benzene acetaldehyde in the A sample is 1.7591, OAV = 2.8950 in the C, and 1.0307 in the G sample. (E,E)-2,4-Decadienal has a strong greasy smell and cucumber aroma, which is usually formed during the automatic oxidation of methyl linoleate oxide (14). Its threshold is extremely low, and it has a high contribution to aroma, the OAV is as high as 146.8601 in C sample. 2-nonenal was described as having fat aroma, wax aroma, cucumber aroma, and melon aroma, which was only detected in A, and OAV was as high as 39.1353, which made an important contribution to the aroma of *F. velutipes*.

#### Phenols

Phenolic compounds generally have a smoky or woody aroma. Butylated hydroxytoluene was detected in A, D, E, and B samples, but their OAVs were low, which made little contribution to the aroma of *F. velutipes*. Phenol has a woody or smoky odor, but its threshold is large (14, 15). It is only detected in G samples in this experiment and its OAV is <1. The OAV in this experimental sample is low, and it does not contribute much to the overall aroma.

#### Acids

In this experiment, only one acid substance was detected, namely acetic acid. Acetic acid has a pungent smell, which was detected in all samples in this experiment, but OAV was <1. This may be caused by microorganisms fermenting different organic substances during the growth and breeding of *F. velutipes*.

#### Esters

Esters are a kind of important aroma compounds, which are mainly formed by the esterification of organic acids and alcohols and have a pleasant fruit aroma (16). A total of 16 ester compounds were detected in 7 different *F. velutipes* varieties in this experiment. Ethyl 2-methylbutyrate has a fruit aroma similar to apple, which is often used to prepare fruit aroma (17). Because of its high content and low threshold, it contributes greatly to the aroma of *F. velutipes*. Ethyl benzoate has a fruit fragrance, exists widely in pineapple, peach, and other fruits, and is often used as food additives (18). The compound was detected in D, E, B, C, F, and G, among which OAV in E, B, and C was >1. Ethyl 2-ethylhexanoate has the aroma of fruits and herbs, and the OAV of it in sample B is 1.9470. Ethyl 2-methylvalerate has a fresh apple-like aroma, and its threshold is only 0.000003, which is only detected in C in this experiment. Ethyl butyrate has a strong apple-and-pineapple-like aroma, but its volatility is strong and its aroma is not lasting (19). It was only detected in F, and its OAV was 8.4394. Ethyl isovalerate (20) has an apple-like fruit aroma, it has an OAV of 872.3671 in the sample F. Isoamyl acetate has a banana-like fruit aroma (13), which is only detected in sample F, and OAV is 12.9788. OAV of other detected esters in 7 *F. velutipes* samples did not exceed 1.

#### Ketones

Total seven ketones, namely, (Z)-geranylacetone, 3-octanone, 2-nonanone, geranylacetone(689-67-8), geranylacetone(3796-70-1), 2-octanone, and 2-heptanone, were detected in all the samples. Ketones are mainly produced by oxidation and degradation of unsaturated fatty acids and degradation of amino acids. The aroma characteristics of 3-octanone were described as fruit aroma (21). It was detected in A, E, C, F, and G, but the OAV was <1, respectively. Fruity, sweet, coconut, and cream smell has been found in 2-Nonanone. Besides the smell of mildew and ketone, 2-Octanone has the smell of milk, cheese, and mushrooms. It was usually added to samples as internal standard for quantitative analysis. Geranyl acetone has a fresh floral aroma with fruit aroma. 2-heptanone has the aroma of spring, fruit, and cinnamon. Yang et al. found 2-heptanone in fermented soybean whey tofu and identified it as the characteristic aroma substance of the product.

#### Ethers

Only one ether substance was detected in this experiment. Anisole has a pleasant smell of fennel, in this study, it was only found in sample F, and the OAV is 0.0057.

#### Hydrocarbons

A total of 19 hydrocarbons were detected in seven *F. velutipes* varieties. D-Limonene is a natural functional monoterpene, which is widely used as an additive of aromas and fragrances in food and has a lemon-like aroma. D-Limonene was detected in all samples, but OAV was > 1 only in D, B, C, and F. D-limonene is a monocyclic monoterpene, which usually exists in the form of a d-isomer and has a fresh orange-like aroma (22). Styrene is a kind of aromatic monomer with slight sweetness, but the smell at high concentration or mixed with other chemicals is unpleasant (23). In this experiment, styrene was detected in all samples except C and G, and its OAV was 1.1959 in sample D and 6.1150 in B. 2,4- decadienal is the product of linoleic acid degradation, which has a pungent aroma, oil aroma, citrus aroma, and chicken aroma. Its aroma threshold is low, which is 0.00004–0.0023 mg/m^3^ in air. It was detected in G and OAV was 3.3830. OAVs of other detected hydrocarbon compounds are <1, which are not described in detail.

#### Others

In this experiment, one sulfide, one acyl compound, and five heterocyclic compounds were detected in the seven *F. velutipes*. Dimethyl trisulfide has a strong cold mint-like smell and a strong spicy fragrance. Natural products exist in fresh onions and canola. Acetoin is an important food spice with pleasant creamy aroma. Pyridine has malodorous smell, which was detected in samples D, E, F, and G, but OAV was small and contributed less to the aroma of *F. velutipes*. The 2-methylpyrazine has the aroma of nut, mildew, and roast, and naturally exists in coffee, white bread, potato chips, almonds, peanuts, fried hazelnuts, fried barley, fried beef, dairy products, and other foods. The 2-pentylfuran has the aroma of bean, fruit, soil, and vegetables-like. The OAV in C is 1.7955 and that in G is 1.3298.

### Partial Least Squares Regression of Seven *F. velutipes* Samples

**Figure 2:**
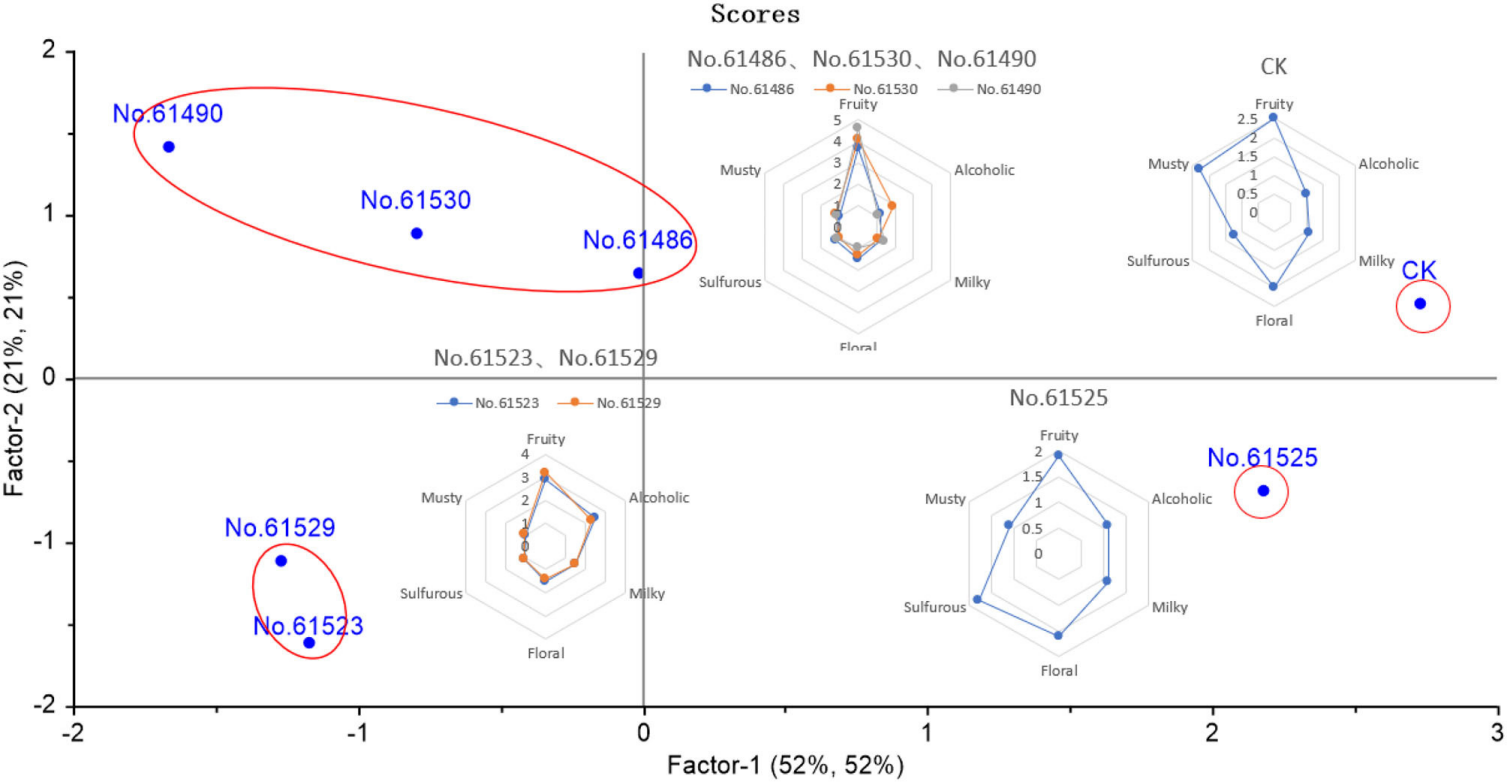
Partial Least Squares Regression (PLSR) based on the OAVs of different compounds of seven kinds of *F. velutipes*.

To explore the relationship between aroma characteristics of different samples, PLSR was performed on seven *F. velutipes* species according to sensory evaluation scores and aroma compounds as Figure 2.

The results of PLSR showed that samples E, C, and F had a high correlation. This result is consistent with the score of sensory evaluation. The three kinds of *F. velutipes* showed strong fruit aroma, and the fruity score of sample F was as high as 4.6, C was 4.1, and 3.1 for E, respectively. This may be due to the high content of ethyl 2-methylbutyrate in all three kinds of *F. velutipes*. As mentioned above, ethyl 2-methyl butyrate has apple-like fruit aroma. There are other compounds that contribute greatly to the fruit aroma, such as ethyl 3-methyl butyrate in F and ethyl 2-methyl valerate in sample C. There is a high correlation between sample A and B. The sensory radar map based on the score of sensory evaluation shows that the aromas of the two kinds of *F. velutipes* are similar. In addition to the fruity aroma, the alcohol of the two kinds of *F. velutipes* also has relatively high-odor intensity; there are milk and floral aroma either. As the detection result of various compounds in the *F. velutipes*, 1-butanol, 3-methyl- has high OAVs in both samples, which may be the reason why they showed relatively high alcoholic aroma. Therefore, phenylacetaldehyde and styrene make an important contribution to the floral aroma of these two kinds of *F. velutipes*. Sample D is different from other samples mainly because of its higher strength of sulfide aroma. This is most likely because of the higher OAV of dimethyl trisulfide in D. And the sample G is mainly different from others because of its strong musty smell. This may be caused by the existence of 2-pentyl furan.

### Venn Analysis

**Figure 3:**
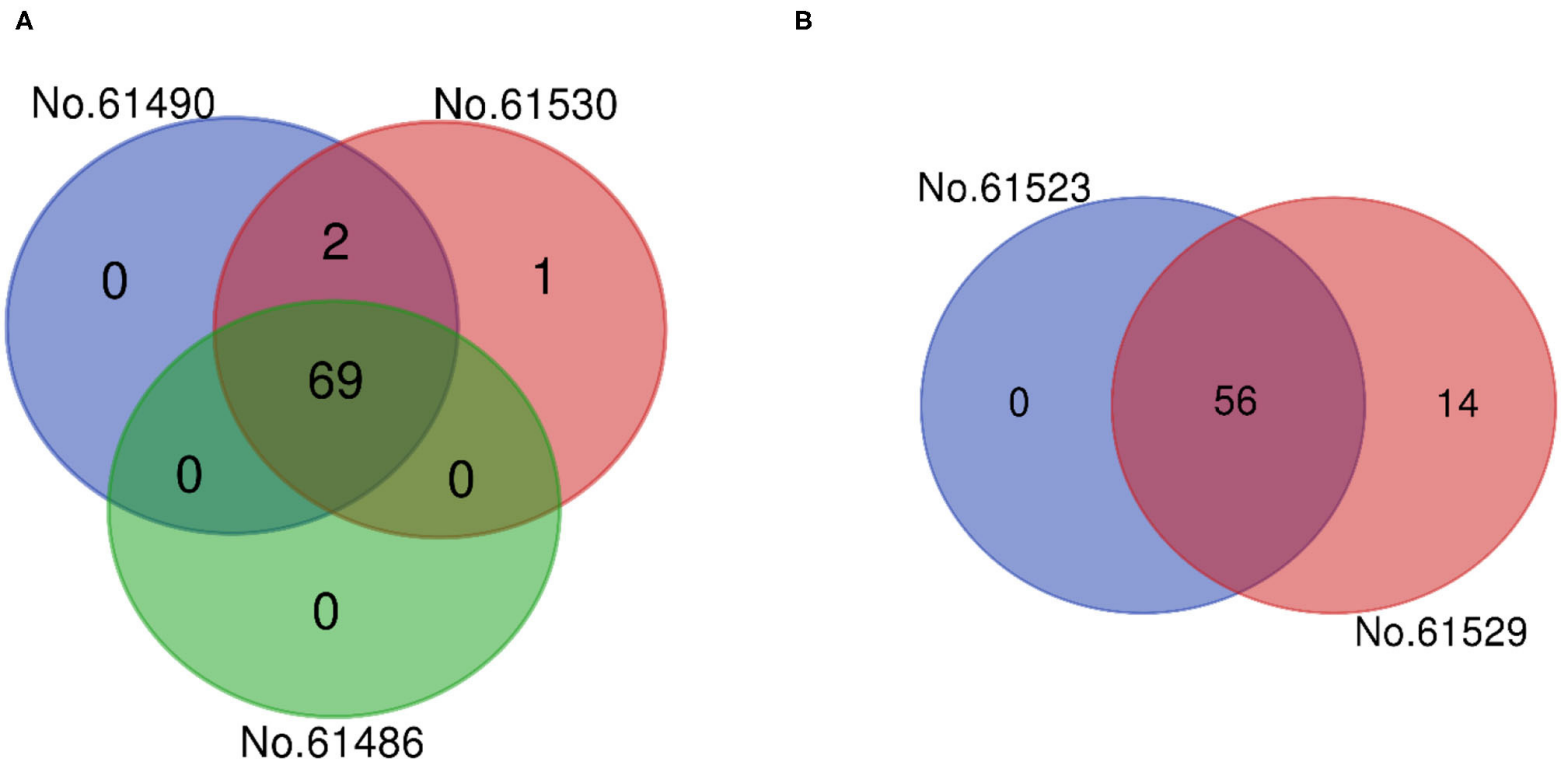
Venn diagram according to the compounds contained in different *F. velutipes* samples. **(A,B)** Represents the strains of samples.

According to Figure 3A, among the three *F. velutipes* samples compared, there were 69 compounds in common among F, E, C, 2 compounds in common between F and E, and only 1 compound in 61,530 alone. This is consistent with the above results of the PLSR analysis. And according to Figure 3B, there were 56 compounds in common among A and D, 14 compounds in D alone. In combination with the above analysis, the *Venn* diagram analysis results showed that the sensory evaluation scores of seven different *F. velutipes* were significantly consistent with the volatile compounds composition.

### Lexicon for Practical Application

Determined by GC-MS analysis, sensory evaluation, and PLSR analysis, the final lexicon is shown in Table 3.

**Table 3:** Lexicon for aroma description of *F. velutipes* studied in this research.

**Aroma Description**		**Volatile Compounds (CAS, Threshold value)**	**Number of Product**
**Aroma**	**Specific**		
**classification**	**description**		
Fruity	Apple-like	Butanoic acid, 2-methyl-, ethyl ester (007452-79-1, 0.000013)
		Butanoic acid, 3-methyl-, ethyl ester (000108-64-5, 0.00001)
		Pentanoic acid, 2-methyl-, ethyl ester (039255-32-8, 0.000003)	No.61523, No.61530, No.61525, No.61486, NO.61490
	Banana-like	1-Butanol, 3-methyl- (000123-51-3, 0.004)
		1-Butanol, 3-methyl-, acetate (000123-92-2, 0.00015)	No.61523, No.61529, No.61530, No.61525, No.61486
			NO.61490, CK
	Cucumber-like	2,4-Decadienal, (E,E)- (025152-84-5, 0.000027)
		2-Nonenal (002463-53-8, 0.00001)	No.61486, No.61523
	Citrus-like	D-Limonene (005989-27-5, 0.034)
		2,4-Decadienal (002363-88-4, 0.0003)	No.61529, No.61525, No.61486, NO.61490, CK
	Berry-like	Ethyl tiglate (005837-78-5, 0.064)	No.61486
Alcoholic	Whisky-like	1-Butanol, 2-methyl- (000137-32-6, 0.0159)
		1-Butanol, 3-methyl- (000123-51-3, 0.004)	No.61523, No.61525, No.61529, No.61530
			No.61486, NO.61490, CK
	Fermented fruit-like	1-Hexanol (000111-27-3, 0.0056)	No.61486
Milky	Creamy-like	Acetoin (000513-86-0, 0.014)	No.61523, No.61525, NO.61490
Floral	Hyacinth-like	Phenylacetaldehyde (000122-78-1, 0.0063)	No.61523, No.61486, CK
	Phoenix-like	Styrene (000100-42-5, 0.065)	No.61529, No.61525
	Iris-like	Hexanoic acid, 2-ethyl-, ethyl ester (002983-37-1, 0.05)	No.61525
	Mint-like	Benzoic acid, ethyl ester (000093-89-0, 0.053)	No.61530, No.61525, No.61486
Sulfurous	Onion-like	Dimethyl trisulfide (003658-80-8, 0.0001)	No.61529
Musty	Mud-like	Furan, 2-pentyl- (003777-69-3, 0.0058)	No.61486, CK

**Table 4:** OAVs of key aroma-active compounds in seven kinds of *F. velutipes*.

**NO**	**CAS**	**Compounds name**	**Threshold values (mg/kg)**	**Concentration (mg/kg)/OAV**						
				**A**	**D**	**E**	**B**	**C**	**F**	**G**
**Alcohol**									
1	000064-17-5	Ethanol	950	1.045 ± 0.012^a^/0.0011	1.900 ± 0.024^a^/0.0020	1.045 ± 0.013^a^/0.0011	2.660 ± 0.021^a^/0.0028	1.615 ± 0.018^a^/0.0017	1.710 ± 0.014^a^/0.0018	0.760 ± 0.009^a^/0.0008
2	000078-83-1	1-Propanol, 2-methyl-	6.5052	0.022 ± 0.002/0.0034	—	—	—	—	—	—
3	000137-32-6	1-Butanol, 2-methyl-	0.0159	0.019 ± 0.004^ab^/1.2256	0.012 ± 0.005^ab^/0.7244	0.008 ± 0.002^ab^/0.5309	0.036 ± 0.008^a^/2.2865	—	—	0.003 ± 0.001^b^/0.2171
4	000123-51-3	1-Butanol, 3-methyl-	0.004	0.047 ± 0.009^ab^/11.8214	0.038 ± 0.006^ab^/9.4962	0.017 ± 0.004^ab^/4.2196	0.099 ± 0.009^ab^/24.7880	0.086 ± 0.005^ab^/21.3854	0.120 ± 0.001^a^/30.0351	0.011 ± 0.002^b^/2.8236
5	000097-95-0	1-Butanol, 2-ethyl-	0.0752	0.002 ± 0.001/0.0275	—	—	—	—	—	—
6	000111-27-3	1-Hexanol	0.0056	0.003 ± 0.001^a^/0.4559	0.004 ± 0.001^a^/0.6946	0.002 ± 0.001^a^/0.4311	—	0.010 ± 0.004^a^/1.8433	—	0.005 ± 0.002^a^/0.8749
7	000589-98-0	3-Octanol	0.078	0.001 ± 0.001^a^/0.0164	—	0.001 ± 0.001^a^/0.0100	—	—	0.001 ± 0.001^a^/0.0125	0.0003 ± 0.0002^a^/0.0040
8	000104-76-7	1-Hexanol, 2-ethyl-	0.3	0.0004 ± 0.0001^a^/0.0012	—	0.0002 ± 0.0001^a^/0.0007	0.004 ± 0.002^b^/0.0118	—	0.0005 ± 0.0002^a^/0.0016	—
9	000628-99-9	2-Nonanol	0.058	0.001 ± 0.001/0.0192	—	—	—	—	—	—
10	000513-85-9	2,3-Butanediol	100	0.0000	—	—	—	—	0.0000	0.0000
11	000060-12-8	Phenylethyl Alcohol	0.56423	0.003 ± 0.002^a^/0.0058	—	—	0.011 ± 0.001^a^/0.0196	0.003 ± 0.001^a^/0.0061	0.021 ± 0.007^a^/0.0368	0.003 ± 0.002^a^/0.0045
12	000123-96-6	2-Octanol	0.0078	—	0.0002 ± 0.0001^a^/0.0272	0.0002 ± 0.0002^a^/0.0272	0.0002 ± 0.0003^a^/0.0272	0.0002 ± 0.0001^a^/0.0272	0.0002 ± 0.0002^a^/0.0272	—
13	000111-87-5	1-Octanol	0.1258	—	0.002 ± 0.001/0.0129	—	—	—	—	—
14	000143-08-8	1-Nonanol	0.0455	—	—	—	0.013 ± 0.005/0.2818	—	—	—
15	000100-51-6	Benzyl alcohol	2.54621	—	—	—	0.003 ± 0.001/0.0010	—	—	—
16	000071-23-8	1-Propanol	8.5056	—	—	—	—	0.016 ± 0.005/0.0019	—	—
17	006032-29-7	2-Pentanol	8.1	—	—	—	—	—	0.0008 ± 0.0003/0.0001	—
18	000626-89-1	1-Pentanol, 4-methyl-	0.82	—	—	—	—	—	0.0003 ± 0.0001/0.0004	—
19	000108-93-0	Cyclohexanol	0.09	—	—	—	—	—	0.0002 ± 0.0001^a^/0.0022	0.0001 ± 0.0001^a^/0.0008
20	007786-67-6	Cyclohexanol, 5-methyl-2-(1-methylethenyl)-	—	—	—	—	—	—	—	—
21	000505-10-2	1-Propanol, 3-(methylthio)-	0.12323	—	—	—	—	—	0.002 ± 0.001/0.0175	—
22	005978-70-1	2-Octanol, (R)-	—	—	—	—	—	—	—	—/0.0052
23	001653-30-1	2-Undecanol	0.0086	—	—	—	—	—	—	0.0002 ± 0.0001/0.0175
**Aldehydes**									
24	000100-52-7	Benzaldehyde	0.75089	0.003 ± 0.002^a^/0.0034	—	0.002 ± 0.001^a^/0.0029	—	—	—	—
25	000122-78-1	Benzeneacetaldehyde	0.0063	0.011 ± 0.004^a^/1.7591	—	—	—	0.018 ± 0.003^a^/2.8950	—	0.007 ± 0.001^a^/1.0307
26	025152-84-5	(E,E)-2,4-Decadienal	0.000077	—	—	—	—	0.011 ± 0.003/146.8601	—	—
27	002463-53-8	2-Nonenal	0.0001	0.004 ± 0.001/39.1353	—	—	—	—	—	—
**Phenols**									
28	000128-37-0	Butylated Hydroxytoluene	1	0.0005 ± 0.0001^a^/0.0005	0.006 ± 0.001^b^/0.0064	0.0006 ± 0.0003^a^/0.0006	0.004 ± 0.002^ab^/0.0040	—	—	—
29	000108-95-2	Phenol	58.58525	0.012 ± 0.004^a^/0.0002	—	—	—	—	0.023 ± 0.003^a^/0.0004	0.0000
**Acid**									
30	000064-19-7	Acetic acid	99	0.030 ± 0.007^a^/0.0003	0.040 ± 0.002^a^/0.0004	0.010 ± 0.005^a^/0.0001	0.059 ± 0.009^a^/0.0006	0.010 ± 0.003^a^/0.0001	0.040 ± 0.009^a^/0.0004	0.010 ± 0.003^a^/0.0001
**Esters**									
31	007452-79-1	Ethyl 2-methylbutyrate	0.000063	0.008 ± 0.002^a^/126.6283	—	0.005 ± 0.001^a^/84.3746	0.103 ± 0.011^b^/1637.6997	0.179 ± 0.015^b^/2835.7906	0.011 ± 0.004^a^/178.1151	—
32	000124-06-1	Tetradecanoic acid, ethyl ester	4	0.008 ± 0.004^a^/0.0021	—	—	—	0.004 ± 0.001^a^/0.0009	0.010 ± 0.005^a^/0.0025	0.002 ± 0.001^a^/0.0005
33	000071-36-3	1-Butanol	0.4592	0.012 ± 0.009/0.0253	—	—	—	—	—	—
34	000112-39-0	Hexadecanoic acid, methyl ester	2	0.012 ± 0.005^a^/0.0058	0.013 ± 0.008^a^/0.0066	0.008 ± 0.002^a^/0.0039	0.020 ± 0.007^a^/0.0097	—	—	—
35	000628-97-7	Hexadecanoic acid, ethyl ester	2	0.049 ± 0.011^a^/0.0244	0.055 ± 0.013^a^/0.0276	0.030 ± 0.004^a^/0.0151	0.041 ± 0.007^a^/0.0207	0.041 ± 0.004^a^/0.0207	0.038 ± 0.005^a^/0.0191	0.015 ± 0.001^a^/0.0073
36	000093-89-0	Benzoic acid, ethyl ester	0.056	—	0.040 ± 0.005^a^/0.7060	0.064 ± 0.014^ab^/1.1489	0.464 ± 0.018^b^/8.2759	0.056 ± 0.011^ab^/1.0061	0.018 ± 0.001^a^/0.3186	0.011 ± 0.003^a^/0.2018
37	000103-36-6	2-Propenoic acid, 3-phenyl-, ethyl ester	0.017	—	0.002 ± 0.001/0.1108	—	—	—	—	—
38	002983-37-1	Ethyl-2-ethylhexanoate	0.05	—	0.002 ± 0.001^a^/0.0447	0.097 ± 0.011^b^/1.9470	0.014 ± 0.004^ab^/0.2815	—	—	—
39	000111-61-5	Octadecanoic acid, ethyl ester	—	—	—	—	—	—	—	—
40	004192-77-2	2-Propenoic acid, 3-phenyl-, ethyl ester, (E)-	0.165	—	—	0.003 ± 0.001^a^/0.0170	0.006 ± 0.003^a^/0.0338	—	0.002 ± 0.001^a^/0.0125	—
41	039255-32-8	Ethyl 2-methylvalerate	0.000003	—	—	—	—	0.002 ± 0.001/700.8519	—	—
42	000105-54-4	Ethyl butyrate	0.0009	—	—	—	—	—	0.008 ± 0.004/8.4394	—
43	000108-64-5	Ethyl isovalerate	0.00001	—	—	—	—	—	0.009 ± 0.003/872.3671	—
44	000123-92-2	Isoamyl acetate	0.00015	—	—	—	—	—	0.002 ± 0.001/12.9786	—
45	000123-66-0	Hexanoic acid, ethyl ester	0.005	—	—	—	—	—	0.003 ± 0.001^a^/0.5044	0.001 ± 0.001^a^/0.1163
46	000106-32-1	Octanoic acid, ethyl ester	0.0193	—	—	—	—	—	0.005 ± 0.002^a^/0.2273	0.002 ± 0.001^a^/0.0771
**Ketone**									
47	003879-26-3	(Z)-Geranylacetone	—	—	—	—	—	—	—	—
48	000106-68-3	3-Octanone	0.0214	0.005 ± 0.001^a^/0.2340	—	0.002 ± 0.001^a^/0.0812	—	0.004 ± 0.002^a^/0.1665	0.002 ± 0.001^a^/0.0996	0.006 ± 0.003^a^/0.2676
49	000821-55-6	2-Nonanone	0.041	—	—	0.001 ± 0.001^a^/0.0198	0.005 ± 0.002^a^/0.1317	—	0.001 ± 0.001^a^/0.0148	—
50	000689-67-8	Geranylacetone	—	—	—	—	0.0000	—	—	—
51	000111-13-7	2-Octanone	0.0502	—	—	—	—	0.008 ± 0.003^a^/0.1622	0.002 ± 0.001^a^/0.0322	0.008 ± 0.004^a^/0.1569
52	003796-70-1	Geranylacetone	0.681	—	—	—	—	0.074 ± 0.013^a^/0.1090	0.092 ± 0.015^a^/0.1352	—
53	000110-43-0	2-Heptanone	0.14	—	—	—	—	—	0.003 ± 0.001/0.0209	—
**Ethers**									
54	000100-66-3	Anisole	0.21	—	—	—	—	—	0.001 ± 0.001/0.0057	—
**Hydrocarbon**									
55	005989-27-5	D-Limonene	0.034	0.005 ± 0.002^a^/0.1551	0.042 ± 0.013^ab^/1.2319	0.015 ± 0.003^ab^/0.4497	0.072 ± 0.009^b^/2.1054	0.147 ± 0.015^b^/4.3074	0.043 ± 0.005^ab^/1.2662	0.028 ± 0.002^ab^/0.8244
56	000100-42-5	Styrene	0.065	0.025 ± 0.009^ac^/0.3818	0.0780.014^bc^/1.1959	0.021 ± 0.007^ac^/0.3270	0.398 ± 0.018^b^/6.1150	—	0.005 ± 0.002^a^/0.0753	—
57	000099-86-5	1,3-Cyclohexadiene, 1-methyl-4-(1-methylethyl)-	0.08	—	0.003 ± 0.001^a^/0.0348	—	—	0.006 ± 0.003^a^/0.0798	—	—
58	000095-47-6	o-Xylene	0.45023	—	0.001 ± 0.001/0.0026	—	—	—	—	—
59	000087-44-5	Caryophyllene	0.064	—	0.017 ± 0.006^a^/0.2633	—	—	—	—	—
60	000091-20-3	Naphthalene	0.006	—	0.003 ± 0.001^a^/0.5346	—	—	—	0.003 ± 0.002^a^/0.5617	—
61	000099-87-6	p-Cymene	0.00501	—	—	0.001 ± 0.001/0.1483	—	—	—	—
62	000108-38-3	Benzene, 1,3-dimethyl-	1	—	—	—	—	0.002 ± 0.001^b^/0.0020	0.001 ± 0.001^ab^/0.0013	0.0002 ± 0.0001^a^/0.0002
63	000555-10-2	.beta.-Phellandrene	0.036	—	—	—	—	0.0003 ± 0.0001/0.0076	—	—
64	000099-85-4	.gamma.-Terpinene	1	—	—	—	—	0.003 ± 0.001/0.0025	—	—
65	007785-70-8	(1R)-2,6,6-Trimethylbicyclo[3.1.1]hept-2-ene	0.0022	—	—	—	—	—	0.001 ± 0.001/0.4458	—
66	013466-78-9	3-Carene	0.14	—	—	—	—	—	—	0.0002 ± 0.0001/0.0014
67	002363-88-4	2,4-Decadienal	0.0003	—	—	—	—	—	—	0.001 ± 0.001/3.3830
**Sulfide**									
68	003658-80-8	Dimethyl trisulfide	0.0001	—	0.001 ± 0.001/10.1895	—	—	—	—	—
**Acyl**									
69	000513-86-0	Acetoin	0.014	0.017 ± 0.004^b^/1.2419	—	0.001 ± 0.001^a^/0.0919	0.028 ± 0.009^b^/1.9762	—	0.017 ± 0.005^b^/1.2246	0.003 ± 0.002^ab^/0.2027
**Heterocyclic**									
70	000110-86-1	Pyridine	2	—	0.003 ± 0.001^a^/0.0016	0.002 ± 0.002^a^/0.0011	—	—	0.003 ± 0.002^a^/0.0014	0.001 ± 0.001^a^/0.0005
71	000109-08-0	Pyrazine, methyl-	30	—	—	—	0.0000	0.003 ± 0.001^a^/0.0001	0.006 ± 0.001^a^/0.0002	—
72	003777-69-3	Furan, 2-pentyl-	0.0058	—	—	—	—	0.010 ± 0.003^a^/1.7955	0.004 ± 0.002^a^/0.6026	0.008 ± 0.003^a^/1.3298
73	070786-44-6	3,6-Dimethyl-2,3,3a,4,5,7a-hexahydrobenzofuran	0.02	—	—	—	—	0.001 ± 0.001^a^/0.0663	—	0.002 ± 0.001^a^/0.0877

*Values bearing different lowercase letters (a, b, c, and d) were significantly different (p ≤ 0.05)*.

Fruit aroma is an important aroma attribute in this experiment, and all samples have fruity aroma in varying degrees. According to the aroma description of different compounds and the intuitive perception of the sensory evaluation panelist, the fruit aroma is divided into apple-like, banana-like, cucumber-like, citrus-like, and berry-like aroma. According to the related compounds and *F. velutipes* listed in the lexicon, *F. velutipes* with a fruity aroma can be used as raw materials in the processing and production of fruit juice beverages and fruity dairy products (24). The alcoholic aroma is mainly contributed by alcohols and divided into whisky aroma and fermented fruit aroma in the lexicon. *F. velutipes* with a mellow aroma may be applied to the production of fruit wine, liquor, and beer through further fermentation. According to aroma characteristics, such as milk, cheese, cream, and *F. velutipes* with milky aroma, can be used in the production of new aroma dairy products.

## Conclusion

To describe the positive and negative aroma attributes of *F. velutipes*, six descriptive terms and their specific characteristics were developed into aroma lexicon. This lexicon is used to describe the aroma characteristics obtained from different varieties of *F. velutipes*. By associating the lexicon with consumer sensory evaluation, these attributes can be associated with specific products. For example, *F. velutipes* with “fruity” aroma can be used in the development of juice products, while alcohol-aromatic varieties can be used in the production of wine products, and creamy varieties can be used in the application of dairy products. In the future, the lexicon could be used in conjunction with consumer acceptance tests of related products to better differentiate consumer definitions of positive and negative aromas in *F. velutipes*. aroma.

## Data Availability Statement

The original contributions presented in the study are included in the article/supplementary material, further inquiries can be directed to the corresponding author/s.

## Author Contributions

RW wrote the original draft. YZ wrote the original draft and made the formal analysis. HL validated the data. JL investigated the study. CS performed data curation. ZX and HY contributed to resources. XS contributed to supervision. TF wrote, reviewed, and edited the manuscript. All authors contributed to the article and approved the submitted version.

## Funding

Funded by Shanghai Agriculture Applied Technology Development Program, China (Grant No.G20200103 - “Enhancement of elite germplasm and promotion of new varieties in industrial edible fungi”). The authors also acknowledge the financial support and fellowship granted by the School of Perfume and Aroma Technology, Shanghai Institute of Technology.

## Conflict of Interest

The authors declare that the research was conducted in the absence of any commercial or financial relationships that could be construed as a potential conflict of interest.

## Publisher's Note

All claims expressed in this article are solely those of the authors and do not necessarily represent those of their affiliated organizations, or those of the publisher, the editors and the reviewers. Any product that may be evaluated in this article, or claim that may be made by its manufacturer, is not guaranteed or endorsed by the publisher.
